# Editorial: Expert opinions and perspectives in complement: 2022

**DOI:** 10.3389/fimmu.2023.1248299

**Published:** 2023-07-12

**Authors:** Marcin Okrój, Nicolas S. Merle, Jinhua Lu

**Affiliations:** ^1^ Intercollegiate Faculty of Biotechnology of University of Gdańsk and Medical University of Gdańsk, Gdańsk, Poland; ^2^ Complement and Inflammation Research Section (CIRS), National Heart, Lung, and Blood Institute (NHLBI), National Institutes of Health (NIH), Bethesda, MD, United States; ^3^ Department of Microbiology and Immunology, Young Loo Lin School of Medicine, National University of Singapore, Singapore, Singapore; ^4^ Immunology Translational Research Programme, Young Loo Lin School of Medicine, National University of Singapore, Singapore, Singapore

**Keywords:** complement inhibitors, COVID-19, SLE, nucleolin, smoking, neuroinflammation, cardiovascular

The complement system is characterized by the numerous and abundant components which are secreted or surface-expressed to mount an effective, regulated, and non-injurious attack on microbial pathogens. By the dawn of the 21^st^ century, the three pathways of complement activation were discovered, i.e. the classical, alternative, or lectin pathways (1). Detailed interfaces between these pathways and known and emerging microbial pathogens continue to reveal new functional aspects of this complex system. In addition, growing numbers of non-microbial complement targets continue to forge new fronts of research such as aging and autoimmunity (2). Intracellular complement activation is yet another new front of complement research that can have broad physiological relevance (3). The complement system is a `double-edged sword’ which eliminates sterile and microbial triggers of tissue inflammation but it amplifies inflammatory damages in excess. In this topic, three major areas of complement functions are discussed.

The complement system is expected to defend against COVID-19 infection but it is probably more noticeable for its contributions to the severe inflammatory tissue injuries observed in advanced patients (4). In the manifestation of complement-mediated damages, the anaphylatoxins resulting from C3 and especially C5 activation, i.e. C3a and C5a, play critical roles (5, 6). In this topic, Stahel and Barnum highlighted the role of complement activation in the pathophysiology of SARS-Cov-2 and other coronavirus infections and brought our attention to the successful use of the C3 inhibitor AMY-101 and the C5 inhibitor eculizumab in severely ill COVID-19 patients. Nonetheless, there is still an unmet need for the complex investigation of complement inhibition as a therapeutic target. The rationale for such studies comes from mouse models showing a milder course of disease upon C3 deficiency or treatment with an antibody that blocks the C5a receptor. Moreover, many studies suggest crosstalk between complement and coagulation cascades and its possible link to thrombotic microangiopathies reported in COVID-19 patients (4). Besides acknowledged inhibitors like AMY-101 and eculizumab that have been tested, several new candidates are considered, including recombinant C1 inhibitor (conestat alpha), another C5 blocker ravulizumab, IFX-1 and advoralimab (monoclonal antibodies blocking C5a-C5aR interaction), and narsoplimab (monoclonal antibody against MASP-2). The latter agent affects the lectin complement pathway, which is the most recently discovered route of cascade activation, thus putatively hiding the most unexplored aspects. Initial components of the lectin pathway are locally produced by alveolar epithelium, a primary target for SARS-CoV-2, and their elevated levels were found in patients’ lung tissue. Colocalization of viral envelope proteins with these markers was also found in blood vessels affected by thrombosis and endotheliitis. Polycarpou et al. describe the resemblance of post-infection Multisystem Inflammatory Syndrome in Children (MIS-C) with Kawasaki disease, an illness of unknown etiology characterized by acute vasculitis in children under 5 years of age. Although the age of patients with MIS-C is typically older, the number of clinical and laboratory parameters in these two syndromes overlap. The authors pointed out that the lectin pathway of complement activation bridges MIS-C and Kawasaki disease, making this complement pathway an attractive target for therapeutic approaches.

Association between the complement system and systemic autoimmune diseases involves most apparently the classical pathway. On the one hand, these autoimmune diseases are hallmarked by immune complexes which deposit in tissues and small blood vessels, and activate the complement pathway through C1q and its associated C1r/C1s proteases (1). It leads to tissue inflammation and injuries which contribute to disease manifestation. Paradoxically, genetic deficiency of the classical pathway (i.e. C1q, C1r, or C1s) often causes systemic lupus erythematosus (SLE), which is a systemic autoimmune disease due to antinuclear autoantibodies (ANA) (7). What causes these pathogenic antibodies remains unclear and, in the article by Wu et al., a comprehensive body of literature has been reviewed to evaluate the nucleolus as a potential ANA trigger and how this may be suppressed by C1q/C1r/C1s.

Like microorganisms, apoptotic cells are also targeted by C1q albeit that C1q binds to apoptotic cells directly without antibodies (8). C1q binds predominantly to the exposed nucleolus (9), which causes C1r/C1s activation and proteolytic degradation of nucleolar autoantigens and alarmins (10–12). Wu et al. divided the nucleus into three distinct regions each representing one clinical ANA staining pattern, i.e. the chromatin network (homogeneous), sites of pre-mRNA processing (speckled), and sites of pre-rRNA processing (nucleolus). They provided documented the structural and functional contexts for these three nuclear regions and discussed autoantigens and alarmins in each of them, hypothesizing that each region is potentially sufficient to induce self-reactive autoantibodies (12–14) and C1q/C1r/C1s dampen their immunogenicity (12, 15).

Previous studies have emphasized the role of the complement system in initiating and propagating a neuroinflammatory response after stroke (16–18). However, while most preclinical stroke models are performed on young healthy animals, they may not be representative of clinical cohorts. In a perspective article, Couch et al. postulate that the inadequacy of *in vivo* stroke models complicates the translation of the beneficial effects observed with multiple neuroprotective agents from mouse to human. Using an *in vivo* model of stroke on aged mice exposed to cigarette smoke, two major co-morbidity factors, authors showed that site-specific inhibition of complement C3 activation with B4-Crry significantly ameliorates neurological deficit by reducing dendritic loss and microglial activation. This study supports that the complement system mediates, at least in part, the neuroinflammatory response in stroke, and might offer a potential alternative treatment to patients who are not eligible for endovascular intervention due to the risk of haemorrhage and oedema.

## Author contributions

JL initiated and coordinated this editorial. MO, NM and JL contributed equally to the content of this editorial. All authors contributed to the article and approved the submitted version.
